# A computational model for sex-specific genetic architecture of complex traits in humans: Implications for mapping pain sensitivity

**DOI:** 10.1186/1744-8069-4-13

**Published:** 2008-04-16

**Authors:** Chenguang Wang, Yun Cheng, Tian Liu, Qin Li, Roger B Fillingim, Margaret R Wallace, Roland Staud, Lee Kaplan, Rongling Wu

**Affiliations:** 1 Department of Statistics, University of Florida, Gainesville, FL 32611 USA; 2 Department of Community Dentistry and Behavioral Science, University of Florida, Gainesville, FL 32611, USA; 3 Department of Molecular Genetics and Microbiology, University of Florida, Gainesville, FL 32611, USA

## Abstract

Understanding differences in the genetic architecture of complex traits between the two sexes has significant implications for evolutionary studies and clinical diagnosis. However, our knowledge about sex-specific genetic architecture is limited largely because of a lack of analytical models that can detect and quantify the effects of sex on the complexity of quantitative genetic variation. Here, we derived a statistical model for mapping DNA sequence variants that contribute to sex-specific differences in allele frequencies, linkage disequilibria, and additive and dominance genetic effects due to haplotype diversity. This model allows a genome-wide search for functional haplotypes and the estimation and test of haplotype by sex interactions and sex-specific heritability. The model, validated by simulation studies, was used to detect sex-specific functional haplotypes that encode a pain sensitivity trait in humans. The model could have important implications for mapping complex trait genes and studying the detailed genetic architecture of sex-specific differences.

## Background

Differences in males and females (sexual dimorphism) is ubiquitous in many biological aspects [1-3]. In humans, sexually dimorphic traits include those from morphological shapes and body size to brain development to disease susceptibility [4,5]. Substantial differences are also observed in sensitivities to pain and pain-killing drugs, and susceptibility to developing chronic pain between men and women [6-8]. All these sex-specific differences are due to varying expression of genes on the X/Y chromosome and autosomes, thought to result from differences in cellular and hormonal environments between the two sexes [9]. A growing body of research has been conducted to elucidate the genetic control of sexual dimorphism in various complex phenotypes by gene mapping approaches [4,5,10,11]. Despite these efforts, however, little is known about the genetic architecture underlying sex-related variation in a quantitative trait.

Since sex is easily determined, the effects of sex on morphological, developmental and pathological traits can be directly observed. However, characterizing the impacts of sex on the genetic architecture of these traits has been challenged by a lack of powerful statistical approaches. The motivation of this article is to develop a statistical and computational model that can systematically search for sex-specific genes contributing to quantitative variation and formulate testable hypotheses regarding the interplay between sexes and gene expression. Our model is principally different from those used in many previous studies that are aimed to detect sex-specific quantitative trait loci (QTLs) based on linkage or linkage disequilibrium analysis [2,4,5,12,13]. Our model will be founded on the statistical framework constructed by Liu et al. [14] to detect the effects and diversity of haplotypes constructed by single nucleotide polymorphisms (SNPs) that are genotyped at candidate genes or genome-wide [15]. Our model has been generalized to allow the test of sex differences in haplotype frequencies, allele frequencies and linkage disequilibria between different SNPs as well as additive and dominant effects of haplotypes on complex traits. It has power to identify sex-specific DNA sequence variants that encode complex phenotypes in men and women.

## Model

### Notation

Suppose there is a diversity of haplotypes constructed by two SNPs each with two alleles designated as 1 and 0. Let *p *and *q *be the 1-allele frequencies for the first and second SNP, respectively. Thus, the 0-allele frequencies at these two different SNPs will be 1 - *p *and 1 - *q*. The two SNPs that are segregating in a natural human population form four haplotypes, [11], [10], [01], and [00], whose frequencies are constructed by allele frequencies and linkage disequilibrium (*D*) between the two SNPs, i.e.,

(1) $$\begin{array}{lll} p_{11} & = & pq+D\\ p_{10} & = & p(1-q)-D\\ p_{01} & = & (1-p)q-D\\ p_{00} & = & (1-p)(1-q)+D \end{array}$$

The parameters contained in equation (1) can be used to describe some important aspects of the genetic structure and diversity of a natural population. Thus, differences in genetic architecture between the two different sexes can be characterized by these sex-specific parameters. Because it is easy to derive the closed forms for estimating haplotype frequencies [14], we will estimate the linkage disequilibrium from the estimated haplotype frequencies.

Let $\Theta_{{\text{M}}_{p}}=(p_{{\text{M}}_{11}},p_{{\text{M}}_{10}},p_{{\text{M}}_{01}},p_{{\text{M}}_{00}})$ and $\Theta_{{\text{F}}_{p}}=(p_{{\text{F}}_{11}},p_{{\text{F}}_{10}},p_{{\text{F}}_{01}},p_{{\text{F}}_{00}})$ be the vectors of haplotype frequencies among males and females, respectively. All the genotypes for the two SNPs are consistent with diplotypes, except for the double heterozygote, 10/10, that belongs to a diplotype of either [11] [00] or [10] [01] (Table 1). Assuming that the population is at Hardy-Weinberg equilibrium, the frequency of a diplotype is expressed as the product of the frequencies of the two haplotypes that construct it. Table 1 characterizes the differences in diplotype frequencies between the males and females.

**Table 1:** Diplotypes and their frequencies for each of nine genotypes at two SNPs, and composite diplotypes for one assumed sex-specific risk haplotype chosen from four possible haplotypes.

	Diplotype			Risk Haplotype in Males				Risk Haplotype in Females			
			
Genotype	Configuration	Male Freq.	Female Freq.	[11]	[10]	[01]	[00]	[11]	[10]	[01]	[00]
11/11	[11] [11]	$p_{{\text{M}}_{11}}^{2}$	$p_{{\text{F}}_{11}}^{2}$	*AA*	$\bar{A}\bar{A}$	$\bar{A}\bar{A}$	$\bar{A}\bar{A}$	*BB*	$\bar{B}\bar{B}$	$\bar{B}\bar{B}$	$\bar{B}\bar{B}$
11/10	[11] [10]	$2p_{{\text{M}}_{11}}p_{{\text{M}}_{10}}$	$2p_{{\text{F}}_{11}}p_{{\text{F}}_{10}}$	$A\bar{A}$	$A\bar{A}$	$\bar{A}\bar{A}$	$\bar{A}\bar{A}$	$B\bar{B}$	$B\bar{B}$	$\bar{B}\bar{B}$	$\bar{B}\bar{B}$
11/00	[10] [10]	$p_{{\text{M}}_{10}}^{2}$	$p_{{\text{F}}_{10}}^{2}$	$\bar{A}\bar{A}$	*AA*	$\bar{A}\bar{A}$	$\bar{A}\bar{A}$	$\bar{B}\bar{B}$	*BB*	$\bar{B}\bar{B}$	$\bar{B}\bar{B}$
10/11	[11] [01]	$2p_{{\text{M}}_{11}}p_{{\text{M}}_{01}}$	$2p_{{\text{F}}_{11}}p_{{\text{F}}_{01}}$	$A\bar{A}$	$\bar{A}\bar{A}$	$A\bar{A}$	$\bar{A}\bar{A}$	$B\bar{B}$	$\bar{B}\bar{B}$	$B\bar{B}$	$\bar{B}\bar{B}$
10/10	$\{\begin{array}{c} \left[11][00\right]\\ \left[10][01\right] \end{array}$	$\{\begin{array}{c} 2p_{{\text{M}}_{11}}p_{{\text{M}}_{00}}\\ 2p_{{\text{M}}_{10}}p_{{\text{M}}_{01}} \end{array}$	$\{\begin{array}{c} 2p_{{\text{F}}_{11}}p_{{\text{F}}_{00}}\\ 2p_{{\text{F}}_{10}}p_{{\text{F}}_{01}} \end{array}$	$\{\begin{array}{c} A\bar{A}\\ \bar{A}\bar{A} \end{array}$	$\{\begin{array}{c} \bar{A}\bar{A}\\ A\bar{A} \end{array}$	$\{\begin{array}{c} \bar{A}\bar{A}\\ A\bar{A} \end{array}$	$\{\begin{array}{c} A\bar{A}\\ \bar{A}\bar{A} \end{array}$	$\{\begin{array}{c} B\bar{B}\\ \bar{B}\bar{B} \end{array}$	$\{\begin{array}{c} \bar{B}\bar{B}\\ B\bar{B} \end{array}$	$\{\begin{array}{c} \bar{B}\bar{B}\\ B\bar{B} \end{array}$	$\{\begin{array}{c} B\bar{B}\\ \bar{B}\bar{B} \end{array}$
10/00	[10] [00]	$2p_{{\text{M}}_{10}}p_{{\text{M}}_{00}}$	$2p_{{\text{F}}_{10}}p_{{\text{F}}_{00}}$	$\bar{A}\bar{A}$	$A\bar{A}$	$\bar{A}\bar{A}$	$A\bar{A}$	$\bar{B}\bar{B}$	$B\bar{B}$	$\bar{B}\bar{B}$	$B\bar{B}$
00/11	[01] [01]	$p_{M_{01}}^{2}$	$p_{F_{01}}^{2}$	$\bar{A}\bar{A}$	$\bar{A}\bar{A}$	*AA*	$\bar{A}\bar{A}$	$\bar{B}\bar{B}$	$\bar{B}\bar{B}$	*BB*	$\bar{B}\bar{B}$
00/10	[01] [00]	$2p_{{\text{M}}_{01}}p_{{\text{M}}_{00}}$	$2p_{{\text{F}}_{01}}p_{{\text{F}}_{00}}$	$\bar{A}\bar{A}$	$\bar{A}\bar{A}$	$A\bar{A}$	$A\bar{A}$	$\bar{B}\bar{B}$	$\bar{B}\bar{B}$	$B\bar{B}$	$B\bar{B}$
00/00	[00] [00]	$p_{M_{00}}^{2}$	$p_{F_{00}}^{2}$	$\bar{A}\bar{A}$	$\bar{A}\bar{A}$	$\bar{A}\bar{A}$	*AA*	$\bar{B}\bar{B}$	$\bar{B}\bar{B}$	$\bar{B}\bar{B}$	*BB*

Two alleles for each of the two SNPs are denoted as 1 and 0, respectively. Genotypes at different SNPs are separated by a slash. Diplotypes are the combination of two bracketed maternally and paternally derived haplotypes. By assuming different haplotypes as a risk haplotype (denoted as *A *for males and *B *for females), composite diplotypes are accordingly defined and their genotypic values are given.

If haplotypes triggers an effect on a quantitative trait, this means that at least one haplotype performs differently from the rest of the haplotypes. Without loss of generality, let haplotype [11] be such a distinct haplotype, called *risk haplotype*, designated as *A*. All the other non-risk haplotypes, [10], [01] and [00], are collectively expressed as $\bar{A}$. The risk and non-risk haplotypes form three *composite diplotypes AA *(symbolized as **2**), $A\bar{A}$ (symbolized as **1**) and $\bar{A}\bar{A}$ (symbolized as **0**). The genotypic values of the three composite diplotypes may be different between the two sexes, arrayed in $\left(\mu_{{\text{M}}_{2}},\mu_{{\text{M}}_{1}},\mu_{{\text{M}}_{0}}\right)$ for the males and $\left(\mu_{{\text{F}}_{2}},\mu_{{\text{F}}_{1}},\mu_{{\text{F}}_{0}}\right)$ for the females, respectively. Let (*a*_M_, *d*_M_) and (*a*_F_, *d*_F_) be the additive and dominance genetic effects due to the risk and non-risk haplotypes in males and females, respectively.

### Likelihoods

Assume that a total of *n *subjects (including *n*_M _males and *n*_F _females) sampled from the population are phenotyped for a quantitative trait. In each sex, there are nine possible genotypes for the two SNPs, each genotype with an observed number generally expressed as $n_{r_{1}{r^{\prime }}_{1}/r_{2}{r^{\prime }}_{2}}^{\text{M}}$ for the males and $n_{r_{1}{r^{\prime }}_{1}/r_{2}{r^{\prime }}_{2}}^{\text{F}}$ for the females (*r*_1 _≥ *r'*_1_, *r*_2 _≥ *r'*_2_, *r*_3 _≥ *r'*_3 _= 1,0).

Many physiological traits scale with body weight (*W*) according to a power function with a certain allometric exponent. Thus, we implement this allometric scaling law to describe the phenotypic value of a trait for subject *i *within male or female subpopulations in terms of the haplotypes considered as

(2) $$\begin{array}{lll} y_{{\text{M}}_{i}} & = & \alpha_{\text{M}}W_{{\text{M}}_{i}}^{\beta_{\text{M}}}+x_{{\text{M}}_{i}}a_{\text{M}}+z_{{\text{M}}_{i}}d_{\text{M}}+e_{{\text{M}}_{i}},\\ y_{{\text{F}}_{i}} & = & \alpha_{\text{F}}W_{{\text{F}}_{i}}^{\beta_{\text{F}}}+x_{{\text{F}}_{i}}a_{\text{F}}+z_{{\text{F}}_{i}}d_{\text{F}}+e_{{\text{F}}_{i}}, \end{array}$$

where (*α*_M_, *β*_M_) or (*α*_F_, *β*_F_) are body weight-related allometric coefficients, $\left(x_{{\text{M}}_{i}},z_{{\text{M}}_{i}}\right)$ or $\left(x_{{\text{F}}_{i}},z_{{\text{F}}_{i}}\right)$ are the indicator variables associated with the additive and dominance effects, respectively, and $e_{{\text{M}}_{i}}$ or $e_{{\text{F}}_{i}}$ is the residual error, normally distributed as $N(0,\sigma_{\text{M}}^{2})$ or $N(0,\sigma_{\text{F}}^{2})$. The genotypic values of composite diplotypes and variance are arrayed by a quantitative genetic parameter vector $\Theta_{{\text{M}}_{q}}=(\alpha_{\text{M}},\beta_{\text{M}},a_{\text{M}},d_{\text{M}},\sigma_{\text{M}}^{2})$ for the males and $\Theta_{{\text{F}}_{q}}=(\alpha_{\text{F}},\beta_{\text{F}},a_{\text{F}},d_{\text{F}},\sigma_{\text{F}}^{2})$ for the females, respectively.

The log-likelihood of haplotype frequencies, genotypic values of composite diplotypes and residual variances given sex-specific phenotypic (**y**_M_, **y**_F_) and SNP data (**S**_M_, **S**_F_) is factorized into two parts, expressed as

(3) $$\begin{array}{ll} & \log L(\Theta_{{\text{M}}_{p}},\Theta_{{\text{M}}_{q}};\Theta_{{\text{F}}_{p}},\Theta_{{\text{F}}_{q}}|y_{\text{M}},S_{\text{M}};y_{\text{F}},S_{\text{F}})\\ = & \log L(\Theta_{{\text{M}}_{p}},\Theta_{{\text{F}}_{p}}|S_{\text{M}},S_{\text{F}})+\log L(\Theta_{{\text{M}}_{q}},\Theta_{{\text{F}}_{q}}|y_{\text{M}},S_{\text{M}},\Theta_{{\text{M}}_{p}};y_{\text{F}},S_{\text{F}},\Theta_{{\text{F}}_{p}}) \end{array}$$

(4) $$\begin{array}{cc} = & \log L(\Theta_{{\text{M}}_{p}}|S_{\text{M}})+\log L(\Theta_{{\text{F}}_{p}}|S_{\text{F}})+\log L(\Theta_{{\text{M}}_{q}}|y_{\text{M}},S_{\text{M}},\Theta_{{\text{M}}_{p}})+\log L(\Theta_{{\text{F}}_{q}}|y_{\text{F}},S_{\text{F}},\Theta_{{\text{F}}_{p}}) \end{array}$$

where equation (4) is derived from equation (3) because the males and females are assumed to be independent, and

(5) $$\begin{array}{ll} \log L(\Theta_{k_{p}}|S_{k})=\text{constant} & \log L(\Theta_{k_{q}}|y_{k},S_{k},\Theta_{k_{p}})=\\ +2n_{11/11}^{k}\log p_{k_{11}} & \sum_{i=1}^{n_{11/11}^{k}}\log f_{k_{2}}(y_{k_{i}})\\ +n_{11/10}^{k}\log(2p_{k_{11}}p_{k_{10}}) & +\sum_{i=1}^{n_{11/10}^{k}}\log f_{k_{1}}(y_{k_{i}})\\ +2n_{11/00}^{k}\log p_{k_{10}} & +\sum_{i=1}^{n_{11/00}^{k}}\log f_{k_{0}}(y_{k_{i}})\\ +n_{10/11}^{k}\log(2p_{k_{11}}p_{k_{01}}) & +\sum_{i=1}^{n_{10/11}^{k}}\log f_{k_{1}}(y_{k_{i}})\\ +n_{10/10}^{k}\log(2p_{k_{11}}p_{k_{00}}+2p_{k_{10}}p_{k_{01}}) & +\sum_{i=1}^{n_{10/10}^{k}}\log[\varphi_{k}f_{k_{1}}(y_{k_{i}})+(1-\varphi_{k})f_{k_{0}}(y_{k_{i}})]\\ +n_{10/00}^{k}\log(2p_{k_{10}}p_{k_{00}}) & +\sum_{i=1}^{n_{10/00}^{k}}\log f_{k_{0}}(y_{k_{i}})\\ +2n_{00/11}^{k}\log p_{k_{01}} & +\sum_{i=1}^{n_{00/11}^{k}}\log f_{k_{0}}(y_{k_{i}})\\ +n_{00/10}^{k}\log(2p_{k_{01}}p_{k_{00}}) & +\sum_{i=1}^{n_{00/10}^{k}}\log f_{k_{0}}(y_{k_{i}})\\ +2n_{00/00}^{k}\log p_{k_{00}} & +\sum_{i=1}^{n_{00/00}^{k}}\log f_{k_{0}}(y_{k_{i}}) \end{array}$$

where $f_{k_{j}}(y_{k_{i}})$ is a normal distribution density function of composite diplotype *j *(*j *= **2**, **1**, **0**) for sex *k*, and

(6) $$\varphi_{k}=\frac{p_{k_{11}}p_{k_{00}}}{p_{k_{00}}p_{k_{00}}+p_{k_{10}}p_{k_{01}}}$$

is the relative proportion of diplotype [11] [00] within the double heterozygote for sex *k*.

It can be seen from equation (3) or (4) that maximizing $L(\Theta_{{\text{M}}_{p}},\Theta_{{\text{M}}_{q}};\Theta_{{\text{F}}_{p}},\Theta_{{\text{F}}_{q}}|y_{\text{M}},S_{\text{M}},y_{\text{F}},S_{\text{F}})$ is equivalent to maximizing log $\left(\Theta_{k_{p}}|S_{k}\right)$ and log $L(\Theta_{k_{p}}|y_{k},S_{k},\Theta_{k_{p}})$ individually in equation (5).

### The EM algorithm

A closed-form solution for the EM algorithm [14] has been derived to estimate the unknown parameters that maximize the two sex-specific likelihoods of (5). The estimates of sex-specific haplotype frequencies are based on the log-likelihood function $\left(\Theta_{k_{p}}|S_{k}\right)$, whereas the estimates of sex-specific genotypic values of composite diplotypes and the residual variance are based on the log-likelihood function $L(\Theta_{k_{p}}|y_{k},S_{k},{\hat{\Theta }}_{k_{p}})$. These two different types of parameters can be estimated using a two-stage hierarchical EM algorithm (see ref. [14] for a detailed implementation).

### Model selection

According to equation (5), the summed likelihood across the sexes, $L(\Theta_{{\text{M}}_{p}},\Theta_{{\text{M}}_{q}}|y_{\text{M}},S_{\text{M}})+L(\Theta_{{\text{F}}_{p}},\Theta_{{\text{F}}_{q}}|y_{\text{F}},S_{\text{F}})$, is formulated by assuming that haplotype [11] is a risk haplotype. However, a real risk haplotype is unknown from raw data (**y**_*k*_, **S**_*k*_). An additional step for choosing the most likely risk haplotype should be implemented. The simplest way to do so is to calculate the likelihood values by assuming that any one of the four haplotypes can be a risk haplotype (Table 1). Thus, we obtain four possible likelihood values as follows:

$$\begin{array}{ccc} & \text{Risk} & \\ \text{No}\text{.} & \text{Haplotype} & \text{Likelihood}\\ 1 & \left[11\right] & L_{1}({\hat{\Theta }}_{{\text{M}}_{p}},{\hat{\Theta }}_{{\text{1M}}_{q}}|y_{\text{M}},S_{\text{M}})+L_{1}({\hat{\Theta }}_{{\text{F}}_{p}},{\hat{\Theta }}_{1{\text{F}}_{q}}|y_{\text{F}},S_{\text{F}})\\ 2 & \left[10\right] & L_{2}({\hat{\Theta }}_{{\text{M}}_{p}},{\hat{\Theta }}_{{\text{2M}}_{q}}|y_{\text{M}},S_{\text{M}})+L_{2}({\hat{\Theta }}_{{\text{F}}_{p}},{\hat{\Theta }}_{2{\text{F}}_{q}}|y_{\text{F}},S_{\text{F}})\\ 3 & \left[01\right] & L_{3}({\hat{\Theta }}_{{\text{M}}_{p}},{\hat{\Theta }}_{{\text{3M}}_{q}}|y_{\text{M}},S_{\text{M}})+L_{3}({\hat{\Theta }}_{{\text{F}}_{p}},{\hat{\Theta }}_{3{\text{F}}_{q}}|y_{\text{F}},S_{\text{F}})\\ 4 & \left[00\right] & L_{4}({\hat{\Theta }}_{{\text{M}}_{p}},{\hat{\Theta }}_{{\text{4M}}_{q}}|y_{\text{M}},S_{\text{M}})+L_{4}({\hat{\Theta }}_{{\text{F}}_{p}},{\hat{\Theta }}_{4{\text{F}}_{q}}|y_{\text{F}},S_{\text{F}}) \end{array}$$

The largest likelihood value calculated is thought to correspond to the most likely risk haplotype. Under an optimal risk haplotype, we estimate sex-specific quantitative genetic parameters ${\hat{\Theta }}_{{\text{M}}_{q}}$ and ${\hat{\Theta }}_{{\text{F}}_{q}}$.

## Hypothesis tests

The genetic architecture of a quantitative trait is characterized by population (including haplotype frequencies, allele frequencies, and linkage disequilibria) and quantitative genetic parameters (including haplotype effects and mode of inheritance for haplotypes). The model proposed provides a meaningful way for estimating the genetic architecture of a trait and further testing sex-specific differences in genetic control.

After haplotype frequencies are estimated, allele frequencies and linkage disequilibrium between the two SNPs with each sex can be calculated as

(7) $$\begin{array}{lll} & \text{Male} & \text{Female}\\ \text{Allele frequency for SNP }1 & p_{\text{M}}=p_{{\text{M}}_{11}}+p_{{\text{M}}_{10}} & p_{\text{F}}=p_{{\text{F}}_{11}}+p_{{\text{F}}_{10}}\\ \text{Allele frequency for SNP }2 & q_{\text{M}}=p_{{\text{M}}_{11}}+p_{{\text{M}}_{01}} & q_{\text{F}}=p_{{\text{F}}_{11}}+p_{{\text{F}}_{01}}\\ \text{Linkage disequilibrium} & D_{\text{M}}=p_{{\text{M}}_{11}}p_{{\text{M}}_{00}}-p_{{\text{M}}_{10}}p_{{\text{M}}_{01}} & D_{\text{F}}=p_{{\text{F}}_{11}}p_{{\text{F}}_{00}}-p_{{\text{F}}_{10}}p_{{\text{F}}_{01}} \end{array}$$

The influence of haplotypes on a quantitative trait is quantified in terms of the additive (*a*) and dominant genetic effects (*d*), and the mode of inheritance (*ρ*), which are estimated for each sex. Each of these population and quantitative genetic parameters can be tested when appropriate hypotheses are formulated.

### Overall genetic control

Haplotype effects on the trait, i.e., the existence of functional haplotypes, in both male and female populations can be tested using the following hypotheses expressed as

(8) $$\begin{array}{ll} H_{0}: & \mu_{M_{j}}\equiv \mu_{M}\text{ and }\mu_{F_{j}}\equiv \text{for }j=2,1,0\\ H_{1}: & \text{At least one equality in }H_{\text{0}}\text{ does not hold} \end{array}$$

The log-likelihood ratio test statistic (LR) under these two hypotheses can be similarly calculated,

(9) $$\text{LR}=-2[\log L_{0}({\tilde{\mu }}_{\text{M}};{\tilde{\mu }}_{\text{F}}|y_{\text{M}},;y_{\text{F}})-\log L_{1}({\hat{\Theta }}_{{\text{M}}_{q}};{\hat{\Theta }}_{{\text{F}}_{q}}|y_{\text{M}},S_{\text{M}},{\hat{\Theta }}_{{\text{M}}_{p}};y_{\text{F}},S_{\text{F}},{\hat{\Theta }}_{{\text{F}}_{p}})],$$

where the *L*_0 _and *L*_1 _are the plug-in likelihood values under the null and alternative hypotheses of (8), respectively. Although the critical threshold for determining the existence of a functional haplotype can be based on empirical permutation tests, the LR may asymptotically follow a *χ*^2 ^distribution with four degrees of freedom, so that the threshold can be obtained from the *χ*^2^distribution table.

### Sex-specific population genetic architecture

The male and female populations may be different in terms of population genetic parameters. Such sex-specific differences can be tested by formulating the following hypotheses

(10) $$\begin{array}{ll} H_{0}: & p_{\text{M}}=p_{\text{F}}\\ H_{1}: & p_{\text{M}}\neq p_{\text{F}} \end{array}$$

for allele frequency at SNP 1,

(11) $$\begin{array}{ll} H_{0}: & q_{\text{M}}=q_{\text{F}}\\ H_{1}: & q_{\text{M}}\neq q_{\text{F}} \end{array}$$

for allele frequency at SNP 2, and

(12) $$\begin{array}{ll} H_{0}: & D_{\text{M}}=D_{\text{F}}\\ H_{1}: & D_{\text{M}}\neq D_{\text{F}} \end{array}$$

for the linkage disequilibrium between the two SNPs.

For each of the hypotheses (10)–(12), the LR values are calculated, which are each thought to asymptotically follow a *χ*^2^-distribution with one degree of freedom. Sex-specific differences in overall population genetic architecture can be tested with the null hypothesis *H*_0_: *p*_M _= *p*_F_, *q*_M _= *q*_F_, and *D*_M _= *D*_F_, with the corresponding LR value to be *χ *^2^-distributed with three degrees of freedom.

### Sex-specific quantitative genetic architecture

Sex-specific differences in overall quantitative genetic architecture can be tested by formulating the hypotheses

(13) $$\begin{array}{ll} H_{0}: & a_{\text{M}}=a_{\text{F}}\text{ and }d_{\text{M}}=d_{\text{F}}\\ H_{1}: & \text{At least one equality in }H_{0}\text{ does not hold} \end{array}$$

The LR value calculated under the null and alternative hypotheses is suggested to follow a *χ*^2^-distribution with two degrees of freedom. The rejection of the null hypothesis implies that the effects of the same haplotype are different between the two sexes. If there exists a sex-specific difference, the next step is to test whether this difference is due to the additive or dominant genetic effects, or both.

### Sex-specific risk haplotypes

In the preceding sections, the same risk haplotype was assumed between the male and female populations. It is possible that the two sexes have different risk haplotypes. Let $\mu_{{\text{M}}_{j_{m}}}$ (*j*_*m *_= **2**, **1**, **0**) and $\mu_{{\text{F}}_{j_{f}}}$ (*j*_*f *_= **2**, **1**, **0**) be the genotypic values of composite diplotypes for the males and females constructed by a sex-specific rick haplotype. By reformulating the likelihood log $L(\Theta_{k_{p}}|y_{k},S_{k},\Theta_{k_{p}})$ of equation (5) based on sex-specific composite diplotypes, these genotypic values can be estimated with the EM algorithm. A best combination of risk haplotypes between the two sexes can be determined from the AIC values.

## Multi-locus haplotyping

### Three-SNP model

Consider three associated SNPs, **S**_1_, **S**_2_, and **S**_3_, each with two alleles denoted by 1 and 0. Let *p*, *q *and *r*, and *D*_12_, *D*_13_, *D*_23 _and *D*_123 _be the 1-allele frequencies for the three SNPs, and the linkage disequilibria between SNPs 1 and 2, SNPs 1 and 3, SNPs 2 and 3 and among the three SNPs, respectively. Eight haplotypes, [111], [110], [101], [100], [011], [010], [001] and [000], formed by these three SNPs, have sex-specific frequencies arrayed in $\Theta_{k_{p}}=(p_{k_{111}},p_{k_{110}},p_{k_{101}},p_{k_{100}},p_{k_{011}},p_{k_{010}},p_{k_{001}},p_{k_{000}})$ for sex *k*. Each of these haplotype frequencies is constructed by allele frequencies at different SNPs and their linkage disequilibria of different orders, expressed as

(14) $$\begin{array}{l} p_{k_{111}}=p_{k}q_{k}r_{k}+p_{k}D_{k_{23}}+q_{k}D_{k_{13}}+r_{k}D_{k_{12}}+D_{k_{123}}\\ p_{k_{110}}=p_{k}q_{k}(1-r_{k})-p_{k}D_{k_{23}}-q_{k}D_{k_{13}}+(1-r_{k})D_{k_{12}}-D_{k_{123}}\\ p_{k_{101}}=p_{k}(1-q_{k})r_{k}-p_{k}D_{k_{23}}+(1-q_{k})D_{k_{13}}-r_{k}D_{k_{12}}-D_{k_{123}}\\ p_{k_{100}}=p_{1}(1-q_{k})(1-r_{k})+p_{k}D_{k_{23}}-(1-q_{k})D_{k_{13}}-(1-r_{k})D_{k_{12}}+D_{k_{123}}\\ p_{k_{011}}=(1-p_{k})q_{k}r_{k}+(1-p_{k})D_{k_{23}}-q_{k}D_{k_{13}}-r_{k}D_{k_{12}}-D_{k_{123}}\\ p_{k_{010}}=(1-p_{k})q_{k}(1-r_{k})-(1-p_{k})D_{k_{23}}+q_{k}D_{k_{13}}-(1-r_{k})D_{k_{12}}+D_{k_{123}}\\ p_{k_{001}}=(1-p_{k})(1-q_{k})r_{k}-(1-p_{k})D_{k_{23}}-(1-q_{k})D_{k_{13}}-r_{k}D_{k_{12}}+D_{k_{123}}\\ p_{k_{000}}=(1-p_{k})(1-q_{k})(1-r_{k})+(1-p_{k})D_{k_{23}}+(1-q_{k})D_{k_{13}}+(1-r_{k})D_{k_{12}}+D_{k_{123}}, \end{array}$$

for sex *k*.

Sex-specific population genetic architecture can be tested by comparing the differences in allele frequencies (*p*_*k*_, *q*_*k*_, *r*_*k*_) and linkage disequilibria of different orders $\left(D_{k_{12}},D_{k_{13}},D_{k_{23}},D_{k_{123}}\right)$ between the males and females.

In a natural population, there are 27 genotypes for the three SNPs. The frequency of each genotype is expressed in terms of haplotype frequencies. Some genotypes are consistent with diplotypes, whereas the others that are heterozygous at two or more SNPs are not. Each double heterozygote contains two different diplotypes. One triple heterozygote, i.e., 10/10/10, contains four different diplotypes, [111] [000] (in a probability of $2p_{k_{111}}p_{k_{000}}$), [110] [001] (in a probability of $2p_{k_{110}}p_{k_{001}}$), [101] [010] (in a probability of $2p_{k_{101}}p_{k_{010}}$) and [100] [011] (in a probability of 2*p*_100_*p*_011_). The relative frequencies of different diplotypes for this double or triple heterozygote are a function of haplotype frequencies (Supporting Information Table 1). The integrative EM algorithm can be employed to estimate the MLEs of haplotype frequencies. A general formula for estimating haplotype frequencies can be derived.

By assuming [111] as a risk haplotype (labeled by *A*) and all the others as non-risk haplotypes (labelled by $\bar{A}$), the formulation of genotypic values for three composite diplotypes, *μ*_2 _for *AA, μ*_1 _for $A\bar{A}$ and *μ*_0 _for $\bar{A}\bar{A}$ can be derived. Similar procedures described for the two-SNP model can be obtained to estimate and test sex-specific additive and dominance genetic effects when a haplotype contains three SNPs.

### *L*-SNP model

It is possible that the two- and three-SNP models are too simple to characterize genetic variants for quantitative variation. We can develop a model that includes an arbitrary number of SNPs whose sequences are associated with the phenotypic variation. A key issue for the multi-SNP sequencing model is how to distinguish among 2^ℓ-1 ^different diplotypes for the same genotype heterozygous at ℓ loci. The relative frequencies of these diplotypes can be expressed in terms of haplotype frequencies.

Consider a a functional haplotype that contains *L *SNPs among which there exist linkage disequilibria of different orders. The two alleles, 1 and 0, at each of these SNPs are symbolized by *r*_1_, ..., *r*_*L*_, respectively. Let $p_{r_{1}}^{k},...,p_{r_{L}}^{k}$ be the allele frequencies for these different SNPs within sex *k*. A haplotype frequency, denoted as $p_{r_{1}r_{2}\cdots r_{L}}$, is decomposed into the following components:

$$\begin{array}{ll} p_{r_{1}r_{2}\ldots r_{L}}^{k} & \\ =p_{r_{1}}^{k}p_{r_{2}}^{k}\ldots p_{r_{L}}^{k} & \text{No LD}\\ +{\left(-1\right)}^{r_{L-1}+r_{L}}p_{r_{1}}^{k}\ldots p_{r_{L-2}}^{k}D_{k_{(L-1)L}}+\ldots +{\left(-1\right)}^{r_{1}+r_{2}}p_{r_{3}}^{k}\cdots p_{r_{L}}^{k}D_{k_{12}} & \text{Digenic LD}\\ +{\left(-1\right)}^{r_{L-2}+r_{L-1}+r_{L}}p_{r_{1}}^{k}\ldots p_{r_{L-3}}^{k}D_{k_{(L-2)(L-1)L}}+\ldots +{\left(-1\right)}^{r_{1}+r_{2}+r_{3}}p_{r_{4}}^{k}\ldots p_{r_{L}}^{k}D_{k_{123}} & \text{Trigenic LD}\\ +\ldots & \\ +{\left(-1\right)}^{L}{\left(-1\right)}^{r_{1}+\ldots +r_{L}}D_{k_{1\ldots L}} & L\text{-genic LD} \end{array}$$

where *D*_*k*_'s are the linkage disequilibria of different orders among particular SNPs for sex *k*.

Sex-specific difference in terms of allele frequencies and linkage disequilibria between different SNPs as well as haplotype additive and dominance effects can be tested by formulating the corresponding hypotheses.

## Results

### Pain genetics study

The model proposed was used to detect differences in the genetic architecture of pain sensitivity between men and women. Genetic and phenotypic data were from a pain genetics project in which 237 subjects (including 143 men and 94 women) from five different races were sampled for six SNPs at three candidate genes. As a demonstration of the utilization of the model, we will focus on two SNPs, OPRDT80G (with two alleles T and G) and OPRDT921C (with two alleles T and C), at the delta opioid receptor. Pain testing procedures followed Fillingim et al. [16]. The phenotypic values of traits were subtracted by the means for each race to remove the effect due to races.

These two SNPs construct four haplotypes, [TC], [TT], [GC], and [GT], which yield 10 diplotypes, [TC] [TC], [TC] [TT], [TT] [TT], [TC] [GC], [TC] [GT], [TT] [GC], [TT] [GT], [GC] [GC], [GC] [GT], and [GT] [GT] and nine genotypes, TT/CC, TT/CT, TT/TT, TG/CC, TG/CT, TG/TT, GG/CC, GG/CT and GG/TT. Based on the observed numbers of each genotype in the male and female populations, we estimated sex-specific haplotype frequencies (Table 1). The pattern of haplotype distribution is consistent between the two sexes, with haplotypes [TC] and [TT] jointly occupying a majority proportion in the populations. Haplotype [GT] is very rare, with the frequency close to zero. SNP OPRDT80G has a low heterozygosity because the frequency of its commoner allele (T) is closer to 0.90, whereas there is a high heterozygosity for SNP OPRDT921C in terms of its averaged allele frequencies. The two SNPs are highly significantly associated at *p *= 3.41 × 10^-5 ^for males and *p *= 9.63 × 10^-5 ^for females, with a normalized linkage disequilibrium of *D' *= 1.00, because alleles T from OPRDT80G and T from OPRDT921C as well alleles G from OPRDT80G and C from OPRDT921C tend to form the same haplotypes more frequently than at random. There is no sex-specific difference in allele frequencies at the two SNPs and their linkage disequilibrium.

**Figure 1:**
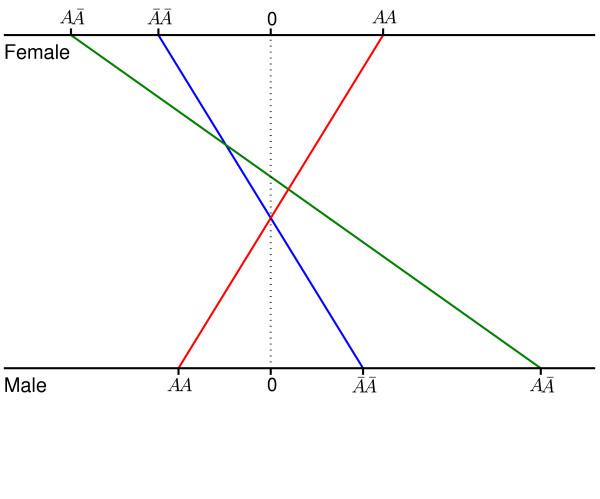
Different genotypic values of baseline pressure pain thresholds measured at the ulna for composite diplotypes, *AA*, $A\bar{A}$, and $\bar{A}\bar{A}$, constructed by risk haplotype [*TT*] and non-risk haplotype in males and females. The origin indicates the mean of the genotypic values between the two homozygotes.

**Figure 2:**
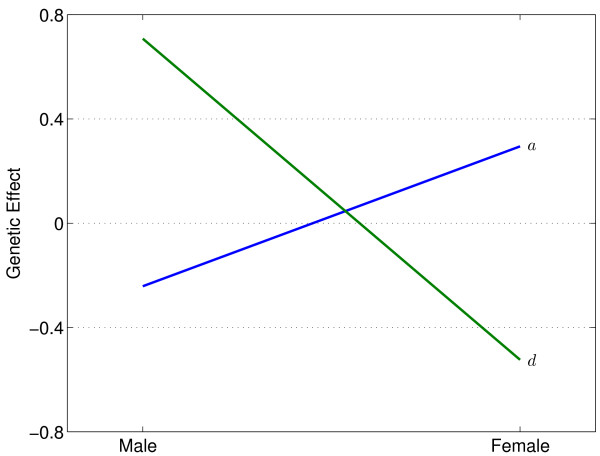
Different additive (*a*) and dominant genetic effects (*d*) of haplotypes on baseline pressure pain thresholds measured at the ulna in males and females.

By assuming that one of the haplotypes is a risk haplotype, we estimated the effects of each haplotype on a pain sensitivity trait, assessed with a baseline pressure pain threshold measured at the ulna, in the pooled male and female population. The likelihoods of haplotype [TC], [TT] and [GC] as a risk haplotype are -594.8, -594.5, and -596.1, and thus the most likely risk haplotype is [TT]. The genotypic values of composite diplotypes constructed by this risk haplotype and its non-risk haplotype counterpart were estimated and compared between different sexes. In both males and female, the three composite diplotypes do not display significant genetic differences in the pain trait studied, but the directions of the additive and dominance effects are different between the two sexes (Table 2). In males, the non-risk haplotype tends to increase pressure pain thresholds, and it is overdominant to the risk haplotype, leading to increased pressure pain thresholds at a marginal significance level (*p *= 0.058) (Fig. 1). By contrast, in females, the non-risk haplotype tends to reduce pressure pain thresholds, and it also tends to be overdominant to the risk haplotype by reducing pressure pain thresholds. These discrepancies in both effect size and direction (Fig. 2) make the overall quantitative genetic architecture of the pain sensitivity trait significantly different between the two sexes (*p *= 1.49 × 10^-7^) (Table 2). Although the additive genetic effect displays a gene by sex interaction at the *p *= 0.03 significance level, a gene by sex interaction for the dominance effect is highly significant at *p *= 6.16 × 10^-7^. No significant difference was observed in inheritance mode between males and females.

**Table 2:** The estimates and tests of population genetic structure for two SNPs, OPRDT80G (with alleles T and G) and OPRDT921C (with two alleles C and T), and quantitative genetic effects of haplotypes constructed by these two SNPs on baseline pressure pain thresholds measured at the ulna in males and females.

	Male		Female			
				
Genetic Parameter	MLE	*p*-value	MLE	*p*-value	Sex-specific LR	Sex-specific *p*-value
**Haplotype Frequency**						
${\hat{p}}_{TC}$	0.364		0.396			
${\hat{p}}_{TT}$	0.505		0.463			
${\hat{p}}_{GC}$	0.131		0.142			
${\hat{p}}_{GT}$	0.000		0.000			
						
**Allele Frequency and Linkage Disequilibrium**						
${\hat{p}}_{T}$ (OPRDT80G)	0.869		0.858		0.079	0.778
${\hat{q}}_{C}$ (OPRDT921C)	0.495		0.537		0.578	0.447
$\hat{D}$	-0.066 3.41 × 10^-5^		-0.066 9.63 × 10^-5^		0.001	0.973
						
**Additive and Dominant Effects and Inheritance Mode for Risk Haplotype [TT]**						
$\hat{a}$ and $\hat{d}$		0.129		0.548	31.44	1.49 × 10^-7^
$\hat{a}$	-0.2	0.431	0.3	0.481	4.72	0.030
$\hat{d}$	0.7	0.058	-0.5	0.350	24.86	6.16 × 10^-7^
$\hat{\rho }$	2.9		1.8		0.63	0.427

### Monte carlo simulation

Simulation studies were performed to test the statistical properties of the model proposed. Given a certain sample size (*n*), we simulated two SNPs by assuming different allele frequencies and linkage disequilibria between two sexes. The hypothesized allele frequencies at the two SNPs are *p*_M _= 0.5, *q*_M _= 0.6 and *D*_M _= 0.1 for males and *p*_F _= 0.8, *q*_F _= 0.9 and *D*_F _= 0.06 for females. By postulating one of the four haplotypes constructed by the two SNPs as a risk haplotype, we calculated the genetic variance among three composite diplotypes using the additive effect *a *= 0.6 and dominance effect *d *= 0.8, from which the residual variance was calculated when the heritability (*H*^2^) of a trait is given. The phenotypic values of the trait were simulated by assuming that they follow a normal distribution under four different simulation designs, (1) *n *= 100 and *H*^2 ^= 0.1, (2) *n *= 400 and *H*^2 ^= 0.1, (3) *n *= 100 and *H*^2 ^= 0.4, and (4) *n *= 400 and *H*^2 ^= 0.4.

The new model was used to analyze the simulated SNP and phenotypic data with the results tabulated in Table 3. Population genetic parameters including allele frequencies (*p *and *q*) and linkage disequilibrium (*D*) can well be estimated, with increasing precision when the sample size increases from 100 to 400. The power to detect the given sex-specific differences in allele frequencies and linkage disequilibrium is as high as 0.95 even with a modest sample size (100). Although the traditional model that does not implement sex-specific differences can provide precise estimates of these parameters, the estimates are generally between the true values of males and females.

**Table 3:** The MLEs of population and quantitative genetic parameters and the standard errors of the estimates obtained by the new model and the power to detect sex-specific differences under different simulation designs. Parameter estimates by a conventional model are also given.

		Sex			
				
Simulation Design	Genetic Parameter	Male	Female	Power	Traditional Model
1	*p*	0.5025 ± 0.0360	0.7829 ± 0.0265	0.95	0.6433 ± 0.0224
	*q*	0.5964 ± 0.0349	0.8732 ± 0.0193	0.95	0.7347 ± 0.0201
	*D*	0.0961 ± 0.0194	0.0181 ± 0.0099	0.80	0.0779 ± 0.0120
	
	*a*	0.6055 ± 0.2008	0.3045 ± 0.1277	0.24	0.4789 ± 0.1192
	*d*	0.3061 ± 0.3371	0.6093 ± 0.1798	0.19	0.5843 ± 0.1875

2	*p*	0.5013 ± 0.0172	0.7980 ± 0.0141	1.00	0.6496 ± 0.0111
	*q*	0.6011 ± 0.0177	0.8958 ± 0.0101	1.00	0.7484 ± 0.0100
	*D*	0.0986 ± 0.0098	0.0084 ± 0.0053	1.00	0.0775 ± 0.0061
	
	*a*	0.6050 ± 0.1114	0.3056 ± 0.0728	0.67	0.4907 ± 0.0616
	*d*	0.2980 ± 0.1604	0.5986 ± 0.0933	0.42	0.5715 ± 0.0966

3	*p*	0.5008 ± 0.0366	0.7835 ± 0.0269	1.00	0.6421 ± 0.0221
	*q*	0.5945 ± 0.0338	0.8731 ± 0.0195	1.00	0.7338 ± 0.0194
	*D*	0.0965 ± 0.0178	0.0161 ± 0.0088	0.86	0.0747 ± 0.0115
	
	*a*	0.5989 ± 0.0924	0.3012 ± 0.0505	0.85	0.4582 ± 0.0483
	*d*	0.2934 ± 0.1352	0.6021 ± 0.0652	0.64	0.5355 ± 0.0715

4	*p*	0.5014 ± 0.0181	0.7974 ± 0.0136	1.00	0.6494 ± 0.0113
	*q*	0.6000 ± 0.0171	0.8968 ± 0.0102	1.00	0.7484 ± 0.0099
	*D*	0.1001 ± 0.0092	0.0077 ± 0.0048	1.00	0.0745 ± 0.0058
	
	*a*	0.6003 ± 0.0439	0.3010 ± 0.0301	1.00	0.4684 ± 0.0251
	*d*	0.3008 ± 0.0659	0.6006 ± 0.0372	0.99	0.5499 ± 0.0360

The estimation of quantitative genetic parameters including the additive (*a*) and dominance effects (*d*) needs the determination of an optimal risk haplotype. When all possible risk haplotypes were assumed for the simulated data, we found that the true risk haplotype gave the largest likelihood among the four possible cases. In general, quantitative genetic parameters can well be estimated, but the estimation precision increases dramatically with sample size and heritability. Although the additive effect can be obtained with reasonable precision at a modest heritability (0.1) with a modest sample size (100), the precise estimation of the dominance effect relies upon a larger heritability and sample size. Also, the given difference in the additive effect between two sexes can be detected with great power, even when both the sample size and heritability are small. But the same size of sex-specific difference in the dominance effect can be detected with the same power only when the sample size is 400 and heritability is 0.4 (Table 3). The traditional model gave biased estimates of the sex-specific additive and dominance effects regardless of increasing sample size and heritability.

We conducted an additional simulation study, in which the data simulated under the assumption of no sex-specific differences in all genetic parameters were analyzed by the new and traditional model. As expected, both the models provide reasonable estimates of population and quantitative genetic parameters, with estimation precision increasing with increasing sample size and heritability (data not shown). This, in conjunction with the results in Table 3, suggests that the new model provides a general tool for study the genetic architecture of a complex trait, regardless of whether the genetic control of the trait is sex-specific.

## Discussion

The genetic architecture of a quantitative trait is complex in terms of interactions between its underlying genetic factors and various environments including sex [1-3]. However, in many current studies, gene by sex interactions are often ignored simply because existing analytical models are not incorporated by environmental factors. While different phenotypic expressions of a trait between the two sexes can be easily measured [4,5], sex-specific discrepancy in the genetic control of the trait can be discerned only when a sophisticated model is used. There is strong evidence for sex-specific genetic influence [17,18] even for traits that display no sexual dimorphism [19]. Although quantitative genetic models are available to estimate sex-specific heritabilities due to aggregative effects of many genes [5,19-22] or map sex-specific QTLs for phenotypic variation [4,5,11-13,23], the model proposed in this article can dissect sex-specific genetic control at the DNA sequence levels.

Our model is founded on the conceptual framework for haplotyping a trait with single nucleotide polymorphisms (SNPs) formulated by Liu et al. [14]. Since haplotypes constructed by physically associated SNPs are thought to affect the expressivity of a complex trait [23,24], it is more precise to characterize such haplotype effects by incorporating gene by gene and gene by sex interactions. Lin and Wu [25] extended Liu et al.'s [14] model to estimate haplotype-haplotype interactions. The new model reported here can not only estimate sex-specific genetic parameters, but also provide a series of statistical procedures for testing sex-specific differences in the genetic architecture of quantitative variation. In a natural population, the structure and pattern of genetic variation can be studied by population genetic parameters, such as haplotype frequencies, allele frequencies and linkage disequilibria. Thus, the understanding of differences in these parameters between the two sexes help to infer the sex-specific genetic structure of a natural population and its evolutionary processes. As shown through simulation studies, our model is alert to discern sex-specific differences in basic population genetic parameters.

Liu et al. [14] assumed a so-called reference or risk haplotype that triggers an effect on complex traits in a different way from the other haplotypes. Thus, the combinations between the risk and non-risk haplotypes (composite diplotypes) will perform differently, depending on the type of combination, i.e., risk by risk, risk by non-risk and non-risk by non-risk. Liu et al. [14] proposed the concepts of the additive effect due to the substitution of the non-risk (or risk) haplotype by the risk (or non-risk) haplotype and the dominance effect due to the interaction between the risk and non-risk haplotypes. These concepts have been integrated into the current model that allows the test of additive by sex and dominance by sex interaction effects. Simulation studies suggest that the new model displays adequate power to detect differences in these quantitative genetic parameters between the sexes, although the detection of sex-specific dominance effects needs a much larger sample size and/or heritability level.

Our model was used to analyze a real data set for pain genetics. Our analyses of a pain sensitivity trait-baseline pressure pain threshold measured at the ulna-by estimating genetic parameters and testing their sex-specific differences in a combination of male and female samples revealed that males and females have different population structure at two SNPs genotyped from a candidate gene (delta opioid receptor) for human pain and that haplotypes exert different genetic effects on the trait between the two sexes. A further test indicates that the risk haplotype [TT] detected by the model exemplifies sex-specific modes of inheritance in affecting the pain trait. While there are no differences among composite diplotypes in males, a significant additive effect was detected in females. Both additive and dominance effects due to the risk haplotype identified are different between the two sexes. Anholt and Mackay [2] described three major mechanisms that explain sex-specific difference in trait control, i.e., sex-specific effects (a gene affects only one sex), sex-biased effects (a gene affects both sexes but to different degrees), and sex-antagonistic effects (a gene affects both sexes but in opposite directions). In our example, the functional haplotype detected affects the pain trait in a sex-antagonistic effect manner, a mechanism thought to help the maintenance of genetic variation in natural populations [26].

In practice, failure to model sex-specific architecture may significantly hamper the ability to detect signals of functional genetic variants in genomewide screens. Although combining male and female data to increase sample size are tempting approaches to increase power, the estimates in this way will be biased from true sex-specific differences. Possible mechanisms that cause sex differences include parent-of-origin effects [27], linkage to or interaction with sex chromosomes, or differences arising from sex-specific hormonal environments. Our gene by sex interaction model that is incorporated by these mechanisms can be modified to consider interactions between genes and any other environments such as life style. Our interaction models should provide a more powerful tool to draw a detailed and precise picture of the genetic architecture of any complex traits that are important to human health.

## Authors' contributions

CW, YC, TL and QL wrote the programs and performed data analyses. RF, MW, and RL designed the experiment. LK performed the experiment. RW conceived the idea and wrote the paper. All authors read and approved the final manuscript.
